# Everything you always wanted to know about the Debye–Waller factor but were afraid to ask

**DOI:** 10.1107/S2052252525004968

**Published:** 2025-06-06

**Authors:** Richard Dronskowski

**Affiliations:** a Institute of Inorganic Chemistry RWTH Aachen University D-52056Aachen Germany

**Keywords:** Debye–Waller factor, thermal motion, anisotropic displacement parameters, computational modeling, molecular crystals

## Abstract

Combining improved diffraction methods, modeling approaches and advanced computations allows for a detailed understanding of atomic thermal motions in crystals. Thus, the Topical Review by Hoser & Madsen [(2025). *IUCrJ***12**, 421–434] covers the Debye–Waller factor, the importance of anisotropic displacement parameters, and the interplay of experiment and theory to accurately capture collective atomic vibrations in molecular crystals.

Although the method of crystal structure determination by diffraction is well over a century old (Friedrich *et al.*, 1913 ▸), its importance continues to grow, and at an increasing rate. We have become accustomed to the fact that every crystalline material is examined sooner or later using diffraction, and ultimately all atomic positions are determined in three-dimensional space, meaning that chemical analysis is also provided free of charge via a detour. Once the atomic positions have been found, quantum-chemical calculations can be carried out, because the sheer existence of the substance, the molecule, the material ultimately needs to be understood. What a wonderful method – quite astonishing. And what would science look like if this method didn’t exist?

Nonetheless, even in a rock-solid crystal the atoms are not standing still, not even at absolute zero temperature (if only for quantum-mechanical reasons, thus spoke Heisenberg). And at finite temperatures the atoms have to move even more, and they have to do so collectively, otherwise it wouldn’t remain a crystal. So collective vibrations in the crystal – called phonons – become excited, and it is precisely these movements that change the X-ray or neutron intensities. Incidentally, contrary to original fears, the moving atoms also result in sharp diffraction images, despite their movement. This is the subject of the topical review by Hoser and Madsen (2025 ▸), two proven experts in this field, in the current issue of *IUCrJ*, using the properly chosen example of small (everyday) molecules.

Their work provides a competent overview of the significance and modeling of thermal movements in crystals. The protagonist is the so-called Debye–Waller factor (Debye, 1913 ▸; Waller, 1923 ▸), which is mathematically attached to the atomic form factor as a correction term. Considering the pure atom–beam interaction, it is precisely the Debye–Waller factor that causes the observed diffraction intensities to decrease at finite temperature due to the atom oscillating back and forth. If the intensities can be measured precisely enough, the thermal movement can also be reconstructed precisely upon modeling these intensities, regardless of which radiation source is used.

Originally it looked – partly due to poor data, partly due to a lack of theory – as if atomic motion were isotropic (*i.e.* the same in all spatial directions). For high-symmetry crystals (cubic table salt is a good example) this crude assumption is sufficient, but not so in more complex cases. In molecular crystals, for instance, the atoms are bound to certain neighboring atoms (but not to others), so the thermal movement can and will be highly anisotropic, because it reflects the differing chemical bondings. Hence, in 2025, we routinely talk about anisotropic displacement parameters (ADPs), which are needed to describe atomic motion mathematically. To do so, a symmetrical 3 × 3 matrix – typically denoted as *U* – is required. Its elements would be very difficult to visualise if C. K. Johnson had not demonstrated, exactly 60 years ago using the ‘Oak Ridge Thermal Ellipsoid Plot’ (*ORTEP*) program, how to represent the thermal movement graphically (Johnson, 1965 ▸). Fig. 1 ▸ shows a typical case in an idealized form. In a figurative sense, we ‘see’ how the atom moves or displaces or oscillates, because the blue atom on the left looks isotropic but as we move towards the right-hand side of the figure the vertical oscillation component (one main axis) becomes increasingly larger, as the quotient for the orange and red atoms indicates. The ellipsoid could of course also be tilted in space, for which the non-diagonal elements of *U* would then be needed.

So much for the gray theory, but life is much more colorful. In the practice of crystal structure analysis, the anisotropic displacement parameters are routinely used to refine a crystal structure as perfectly as possible, under the plausible assumption – but what an assumption! – that these ADPs actually describe the atomic motions correctly. However, it is also conceivable that the ADPs conceal or disguise certain residual errors or even incorrect models, as ‘botch’ parameters, so to speak. So do the ADPs actually reflect the thermal motion? Or is the structural model incorrect, possibly disordered? How can one demonstrate that? Could powerful theory help, especially in experimentally difficult situations?

Using small molecules as the most fitting examples, Hoser and Madsen discuss various approaches to modeling the atomic thermal motion in a very pedagogical way, starting with lattice dynamics and dispersion analysis of various phonon modes in reciprocal space. They continue with the more-or-less direct calculation of the Debye–Waller factor using (empirical) force fields, *ab initio* calculations and molecular dynamics simulations. A real challenge lies in the *anharmonic* movements that lead to, for example, thermal expansion of the crystal, and which can be described using extended formulas (say, Gram–Charlier series). High-resolution diffraction data as complete as possible are essential here, in addition to a correct structural (and ideally also electronic structure) model. The challenges of hydrogen atoms and the comparability of X-ray and neutron diffraction are also explained, including approaches for validating ADPs.

In their review, Hoser and Madsen finally discuss specialized models (*e.g.* ‘rigid body’ and ‘TLS’ = translation/libration/screw analysis) that allow the interpretation of collective motions of groups of atoms belonging together, and they explain the *SHADE* approach for the treatment of hydrogen atoms bonded to C, N and O. In addition, thermodynamic quantities such as vibrational entropies or heat capacities can be estimated from really precise ADPs. All this results in a close interplay between precise experiments, advanced theory and computer simulations, these days known as ‘quantum crystallography’, to capture precisely thermal motion (and possible deviations from that) in small-molecule crystals. In the future, diffuse scattering will also be looked at more closely, and machine learning will be given its chance.

That being said, a lot has happened since 1913, and in view of more precise measurements and – the subject of their topical review – considerably more powerful modeling methods, nothing stands in the way of the quantitative analysis of atomic movements. There is a great deal of additional information in these movements that is worth exploiting, as nicely demonstrated by Hoser and Madsen.

## Figures and Tables

**Figure 1:**
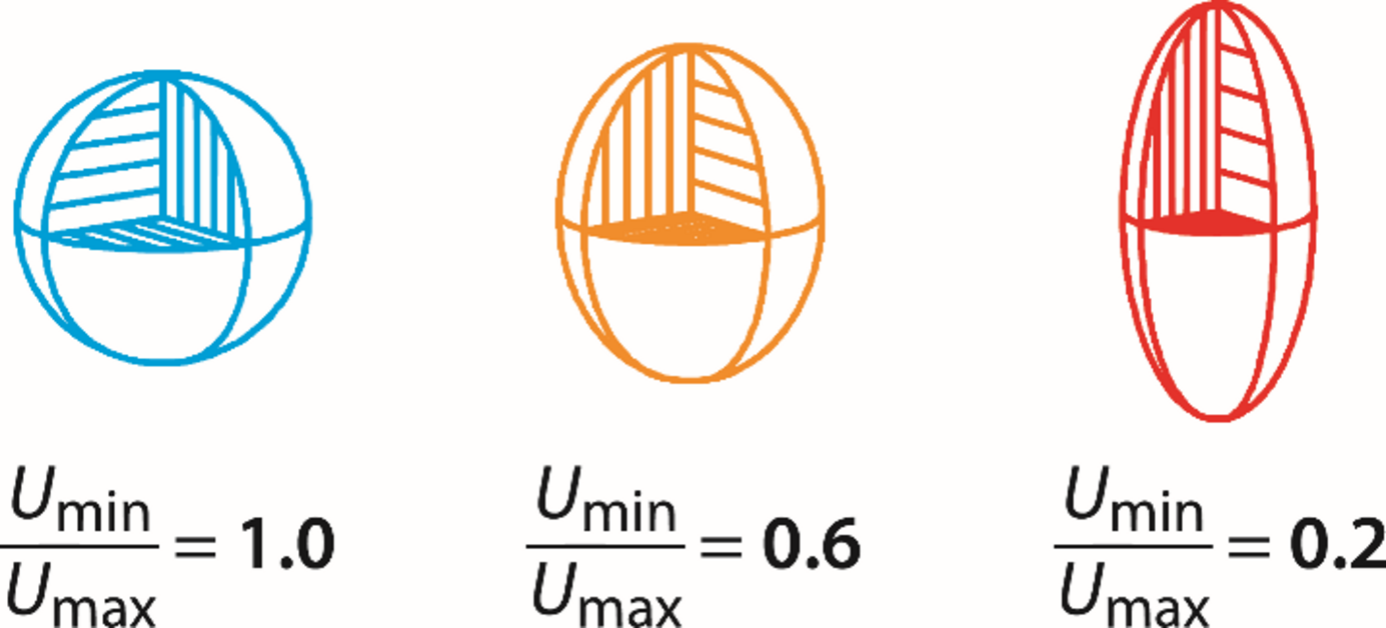
An *ORTEP* representation of an arbitrary atom with different ratios of lowest and highest principal-axis displacement parameters, *i.e.* reflecting the amount of asphericity, with most isotropic on the left (in blue) and most anisotropic on the right (in red). Adapted from Deringer *et al.*, 2014 ▸, with permission from The Royal Society of Chemistry.

## References

[bb1] Debye, P. (1913). *Annalen Phys.* **348**, 49–92.

[bb2] Deringer, V. L., Stoffel, R. P., Togo, A., Eck, B., Meven, M. & Dronskowski, R. (2014). *CrystEngComm* **16**, 10907–10915.

[bb3] Friedrich, W., Knipping, P. & Laue, M. (1913). *Annalen Phys.* **346**, 971–988.

[bb4] Hoser, A. & Madsen, A. Ø. (2025). *IUCrJ* **12**, 421–434. 10.1107/S2052252525004361 PMC12224083 40476304

[bb5] Johnson, C. K. (1965). *ORTEP: A Fortran Thermal-Ellipsoid Plot Program for Crystal Structure Illustrations*. Report ORNL-3794 (Rev.), Union Carbide Corp. and Oak Ridge National Laboratory, Tennessee, USA.

[bb6] Waller, I. (1923). *Z. Phys.* **17**, 398–408.

